# Probabilistic mapping of tremor control and gait ataxia risk in deep brain stimulation

**DOI:** 10.1007/s00702-026-03137-9

**Published:** 2026-03-26

**Authors:** Benedikt Weigl, Regina Pistorius, Jonas Roothans, Nicolò G. Pozzi, Steffen Paschen, Günther Deuschl, Jens Volkmann, Martin M. Reich

**Affiliations:** 1 Department of Neurology, University Hospital and Julius-Maximilian-University, Josef-Schneider-Str. 11, D-97080 Wuerzburg, Germany; 2 Department of Neurology, University Hospital and Christian-Albrechts-University, Kiel, Germany

**Keywords:** Essential tremor, Deep brain stimulation, Probabilistic stimulation mapping, Gait ataxia

## Abstract

Deep brain stimulation (DBS) is an effective therapy for medication-refractory essential tremor, yet clinical outcomes remain highly variable and stimulation-induced side effects, particularly gait ataxia, frequently limit long-term benefit. In routine clinical practice, it is difficult to predict which stimulation settings will provide optimal tremor control while avoiding disabling motor complications. We analysed long-term tremor and gait outcomes in 73 patients from two DBS centres with chronic bilateral (sub)thalamic DBS, assessed before surgery and at follow-up after 12 months using blinded ratings of standardized video recordings. We evaluated the influence of demographic, clinical, and stimulation parameters and applied voxel-wise probabilistic stimulation mapping to identify anatomical regions associated with tremor suppression and stimulation-induced gait ataxia. DBS produced a marked reduction in tremor (46.3% ± 4.3), with 77% of patients free of functionally disabling tremor (> 2 cm amplitude) at follow-up, but 44% exhibiting clinically relevant gait ataxia. No demographic, clinical, or stimulation parameter was independently associated with tremor improvement. However, probabilistic mapping revealed a tremor “sweet spot” just superior to the medial subthalamic nucleus, and stimulation field–based modelling predicted individual tremor outcome in leave-one-out cross-validation, explaining 21% of variance. In contrast, higher stimulation amplitudes and higher age at surgery were associated with greater stimulation-induced gait worsening, which localized to posterior–inferior stimulation in the subthalamic area. Integrating these factors enabled leave-one-out cross-validated prediction of gait outcome, explaining 49% of variance. Together, these results show that the clinical variability of DBS in essential tremor reflects both spatial targeting and patient-specific susceptibility. Individualized prediction models based on probabilistic stimulation mapping can integrate these factors to estimate stimulation outcome.

**Supplementary Information:**

The online version contains supplementary material available at https://doi.org/10.1007/s00702-026-03137-9.

## Introduction

Essential tremor (ET) is one of the most common movement disorders and often remains disabling despite optimized medical treatment (Louis et al. 2010). Deep brain stimulation (DBS) of the (sub)thalamic region is an established therapy even in medication-refractory ET and has been shown to lead to marked tremor reduction (Benabid et al. 1991).

While mean tremor scores are reduced by about 60% following DBS, study outcomes span a wide range from minimal to near-complete tremor suppression and individual patient responses are difficult to predict, with notably poorer responses of axial (head and voice) compared with appendicular tremor (Lu et al. 2020; Hopfner and Deuschl 2020). In the long-term, habituation to DBS may further mitigate benefit (Paschen et al. 2019; Fasano and Helmich 2019).

Moreover, stimulation-induced side effects such as dysarthria, paresthesias, and gait imbalance or ataxia are common and can often be therapy-limiting, and therefore require active consideration in clinical practice (Martinez-Nunez et al. 2024). Among these, stimulation-induced ataxia has gained particular attention as it may occur in up to 35% of treated patients (Chiu et al. 2020). A particularly intriguing clinical side effect is a form of delayed-onset gait ataxia that may only emerge after prolonged periods of stimulation, making it difficult to anticipate and account for during routine programming (Reich et al. 2016; Kim et al. 2021). In one case, this syndrome developed rapidly after five years of chronic stimulation but was reversible following a change in stimulation parameters (Contarino et al. 2017). Such cases highlight the need to identify stimulation settings and patients at risk in order to avoid long-term complications while optimizing tremor control.

However, anatomically disentangling therapeutic from adverse effects in the subthalamic region is inherently difficult as multiple cerebello-thalamo-brainstem pathways converge within a small volume, creating overlapping benefit and side-effect territories. In addition, pathway-specific sensitivity to stimulation means that similar contact locations can produce divergent clinical effects, which may explain why outcome prediction has been more successful for tremor suppression but so far not for stimulation-induced side effects (Dembek et al. 2017; Levy et al. 2020). These uncertainties are reflected in the ongoing debate over the optimal anatomical target for tremor alleviating DBS, with stimulation sites including the ventral intermediate nucleus of the thalamus (VIM) to the posterior subthalamic area (PSA) or zona incerta (ZI) (Ruegge et al. 2020; Sandvik et al. 2012).

While neuro- and nuclear imaging approaches are able to characterize DBS network effects (Younger et al. 2023; Honkanen et al. 2024), probabilistic stimulation mapping has emerged as a complementary approach to disentangle local stimulation effects by analysing volumes of tissue activated (VTA) in common anatomical space (Butson et al. 2011). Initially developed to identify so called “sweet” and “sour” spots for surgical targeting, probabilistic mapping has since been extended to generate individual-level outcome predictions from each patient’s stimulation field, enabling more fine-grained risk–benefit estimations and even model-based reprogramming (Reich et al. 2019; Lange et al. 2023, 2025b).

As such analyses rely only on routine clinical imaging and chronic stimulation settings, they enable the leveraging of large retrospective cohorts. When combined with quantitative score-based phenotyping focussed on specific symptoms, this provides an opportunity to disentangle the contributions of patient characteristics, clinical state, and stimulation parameters, together with their specific local stimulation effects, for both tremor control and stimulation-induced gait ataxia.

The present work aims to disentangle regions mediating tremor control from those driving stimulation-induced gait ataxia and to evaluate their value together with demographic and stimulation factors for predicting individual clinical benefit and risk.

## Methods

### Subjects

This retrospective study included patients with pharmacoresistant bilateral tremor who underwent bilateral (sub)thalamic deep brain stimulation of the subthalamic area at two centres following previously described surgical procedure (implanted within 2003–2015 in Kiel and within 2009–2017 in Würzburg)(Herzog et al. 2007). Inclusion criteria were: (i) diagnosis of essential tremor according to the Movement Disorder Society Consensus Statement (Deuschl et al. 1998); (ii) stable clinical response under DBS for at least 6 months with the chronic stimulation parameters; (iii) video documentation of the clinical motor state within 6 months before surgery and at follow-up after 12 months; (iv) pre- and postoperative imaging suitable for lead reconstruction.

### Clinical evaluation and statistical analysis

Tremor severity and ataxia were assessed from preoperative and long-term follow-up videos rated by a blinded movement disorder specialist (NGP). Tremor severity was quantified using the Fahn–Tolosa–Marin Tremor Rating Scale (TRS) (Fahn et al. 1993), and gait disturbances were rated using items 1–3 of the Scale for the Assessment and Rating of Ataxia (SARA) (Schmitz-Hubsch et al. 2006). As primary outcome variable tremor was expressed as both absolute and relative improvement while ataxia outcomes were expressed as absolute changes from baseline. Changes in tremor and gait were analysed using paired two-sided Wilcoxon signed-rank tests on within-subject differences following a Shapiro-Wilk test for normality. In addition, we applied a score-independent categorization to identify patients with clinically relevant disability based on tremor amplitude (> 2 cm) in any extremity during postural or intention tremor, assessed pre- and postoperatively (Friedrich et al. 2024).

For the gait ataxia outcome, patients were stratified a priori into clinically relevant subgroups based on SARA items 1–3 at follow-up. Patients with scores ≤ 3 were classified as the “preserved gait” group, showing little to no observable ataxia. Those with scores > 3 were categorized as the “gait disturbance” group, exhibiting emerging ataxic symptoms. Patients with severe gait impairment (SARA_1–3_ ≥7) formed a “severe gait ataxia” subgroup. Category distribution changes from pre- to active DBS were analysed using a Stuart–Maxwell test for marginal homogeneity.

We then assessed the influence of demographics and baseline clinical conditions (age at surgery, disease duration, baseline TRS and SARA scores) and stimulation parameters (amplitude, pulse width, frequency, averaged across both contacts in each patient) on tremor improvement and the emergence of ataxia. First, univariable associations were examined using Spearman correlations between each predictor and both relative tremor improvement and ataxia outcomes. As these correlations do not account for multiple comparisons and do not allow direct comparison of effect sizes, all predictors were subsequently entered into multivariable linear regression models to estimate standardized β-coefficients and identify independent predictors reporting overall model significance and multivariate adjusted R^2^. Variance inflation factors were calculated to assess potential multicollinearity among predictors.

### Lead localisation and stimulation field modelling

Electrode positions were reconstructed in individual patient space using SureTune3™ (Medtronic) by fusing preoperative isotropic T1-MRI (1 mm^3^) with postoperative CT (0.625 × 0.625 × 1 mm) or, if unavailable, postoperative isotropic MRI. Volumes of tissue activated (VTAs) were then estimated for each lead based on the individual stimulation settings using the standard settings of the implemented biophysical model (Astrom et al. 2009). Basal ganglia structures, including the subthalamic nucleus (STN), red nucleus (RN), and substantia nigra (SN), were segmented on the preoperative MRI. As in previous work these segmentations were used as anatomical references in the Arena pipeline to nonlinearly normalize electrodes and VTAs to common MNI space (ICBM 2009b NLIN asymmetric) (Navratil et al. 2025). Right-hemisphere VTAs were mirrored onto the left hemisphere nonlinearly. This standard approach in DBS mapping enables anatomically consistent, hemisphere-independent heatmaps, at the cost of potential lateralized effects.

### Spatial analysis, heatmap creation and probabilistic mapping of stimulation outcome

Following our previous work, we used a probabilistic mapping approach to characterize the spatial distribution of tremor and ataxia effects within the stimulated region (Reich et al. 2019; Nickl et al. 2025; Navratil et al. 2025). In short, for each (0.25 × 0.25 × 0.25 mm) voxel in MNI template space, patients whose VTAs included that voxel were compared with those whose VTAs did not using voxel-wise two-sample t-tests, retaining the sign of the t-value. The resulting signed p-maps indicated whether stimulation at each voxel was associated with improvement or worsening of tremor or gait ataxia.

For anatomical reference and spatial comparison, we defined a tremor-alleviation “sweet” spot and a gait-ataxia “sour” spot as clusters in these maps where stimulation was significantly associated with greater tremor improvement or greater gait worsening (*p* < 0.05, minimum cluster size 50 voxels (Navratil et al. 2025). Next, we applied a non-parametric combination (NPC) analysis across both outcome contrasts using FSL-PALM (standard settings; 5,000 permutations), to identify a joint anatomical target for optimal tremor control with minimal gait ataxia using the same cluster thresholds (Winkler et al. 2014, 2016). The centre of gravity (COG) of all clusters was computed as the mean 3-D coordinate of all its voxels and reported in AC-PC reference space using the Arena pipeline.

As a first spatial analysis, we related tremor and gait ataxia outcomes to the average Euclidean distance between each patient’s effective “active contact”, defined as the COG of the individual VTA, and the COG of the corresponding sweet or sour spot using a Spearman correlation.

To then create and test a clinical prediction model based on probabilistic mapping, voxel values from the signed p-maps overlapping each patient’s VTA were extracted and summarized in 15-bin histograms representing the distribution of beneficial versus detrimental heatmap values within that patient’s stimulation field. After an initial correlation analysis, these histograms served as predictors in multivariable linear regression models trained using leave-one-out cross-validation. For each iteration, the model was trained on all but one patient and used to estimate the left-out patient’s tremor or gait outcome based solely on stimulation location. Model performance was quantified by correlating predicted and observed clinical scores across patients, thereby evaluating the predictive utility of spatial stimulation mapping in ET (Navratil et al. 2025).

We additionally augmented these spatial prediction models with the independent predictors previously identified from the multivariable clinical analysis as a final outcome model, again checking for multicollinearity using VIF.

## Results

### Clinical characteristics

Overall, 73 patients (38 male) with chronic bilateral stimulation of the subthalamic area fulfilling the inclusion criteria for essential tremor and complete video, imaging, and stimulation data were included. The mean age at surgery was 65.6 ± 1.2 years (mean ± SEM), with an average disease duration of 32.9 ± 2.2 years prior to DBS. Changes in both clinical outcome variables deviated from normality (Shapiro–Wilk test), with *p* = 0.011 for tremor and *p* < 0.001 for ataxia. Tremor improved significantly after DBS, with Tremor Rating Scale scores decreasing from 23.3 ± 1.1 to 12.5 ± 1.0 (Wilcoxon *p* < 0.0001), corresponding to a mean tremor improvement of 46.3% ± 4.3%. From a clinical perspective, 55 of 73 patients (75%) exhibited postural or intention tremor with functionally relevant, disabling amplitudes > 2 cm in at least one extremity prior to DBS, whereas only 17 patients (23%) showed such amplitudes under active stimulation.

In contrast, ataxia scores (SARA) worsened significantly, increasing from 1.2 ± 0.2 before DBS to 3.0 ± 0.3 under stimulation (Wilcoxon *p* < 0.0001). Regarding clinically relevant gait changes, before DBS most patients exhibited little to no gait impairment (SARA_1–3_ ≤ 3; *n* = 67) or mild gait disturbance (SARA_1–3_ = 3–6; *n* = 6), with no cases of severe ataxia. Under stimulation, gait impairment increased, with 24 patients exhibiting gait disturbance and 8 patients developing de novo severe gait ataxia (SARA_1–3_ ≥7). Accordingly, the overall distribution of gait severity categories shifted significantly under DBS (Stuart–Maxwell test for marginal homogeneity: χ² = 25.7, *p* < 0.0001).

**Fig. 1:**
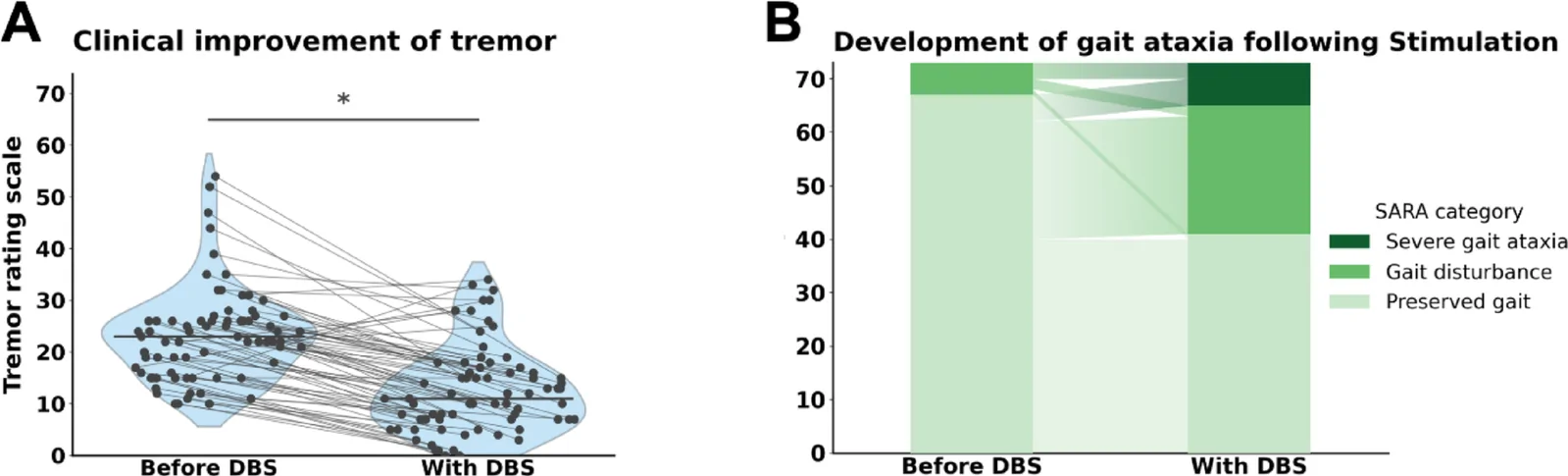
Stimulation effects on tremor and the emergence of Ataxia. A: DBS led to a significant improvement of tremor severity scores 23.3 ± 1.1 to 12.5 ± 1.0 (Wilcoxon *p* < 0.0001) with a mean tremor improvement of 46%. B: While just 6 patients showed mild gait disturbance before DBS, stimulation led to 24 patients developing such gait disturbances and a further 8 patients developing severe gait ataxia

Overall, while DBS effectively suppressed tremor, it was also associated with a marked worsening of ataxia, resulting in 44% (*n* = 32) of patients developing clinically noticeable gait difficulties (Fig. 1).

### Determinants of tremor and stimulation-induced ataxia

Univariable analyses showed no association of cohort demographics or baseline clinical conditions with tremor outcome, with neither age at surgery (Spearman ρ = −0.10), disease duration (ρ = 0.10), baseline tremor severity (ρ = −0.13), nor baseline ataxia (ρ = −0.03) showing a significant correlation with relative tremor improvement. With regards to stimulation parameters, higher stimulation amplitude and longer pulse width were associated with less relative tremor improvement (ρ = −0.25, *p* = 0.033; ρ = −0.24, *p* = 0.041), whereas stimulation frequency showed no association (ρ = 0.04). While these associations were attenuated in the multivariable model, none of these predictors demonstrated a statistically significant independent effect. In other words, tremor improvement was not strongly determined by any of the included clinical or stimulation parameters, and the model of all clinical and stimulation predictors overall did not predict tremor outcome (adjusted R² = 0.07, *p* = 0.1).

When examining stimulation-induced ataxia, univariable correlations showed significant positive associations with older age at surgery (ρ = 0.28, *p* = 0.016) whereas neither disease duration (ρ = −0.12), baseline tremor severity (ρ = −0.10) or notably baseline ataxia (ρ = −0.04) showed significant associations. Concerning stimulation parameters, higher stimulation amplitudes were the only parameter associated with worsening of gait scores (ρ = 0.34, *p* = 0.004) with frequency (ρ = −0.08) and pulse width (ρ = 0.21) showing no significant association. In the multivariable model, age at surgery and stimulation amplitude remained statistically significant predictors for ataxia outcome. In addition, baseline tremor severity and stimulation frequency emerged as significant negative predictors after accounting for the other predictors. Pulse width, while showing a positive trend did still not reach significance in the multivariate model (*p* = 0.066). Overall, this model was a modest predictor for change in SARA score (adjusted R² = 0.29, *p* < 0.001). Variance inflation factors were low across all predictors (VIF < 1.5), indicating that multicollinearity did not meaningfully influence these estimates.

**Fig. 2:**
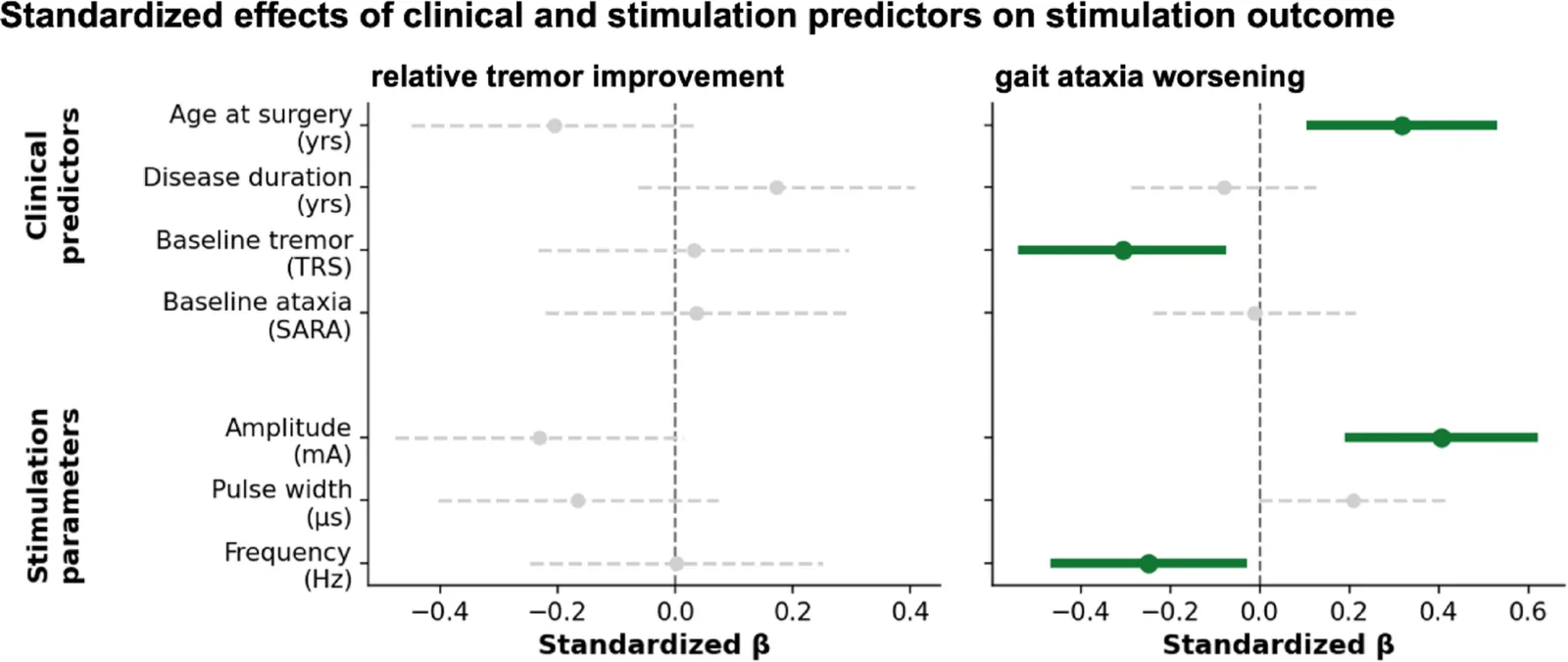
Effects of clinical and stimulation predictors on tremor improvement and gait ataxia. Forest plots of standardized beta coefficients and 95% model confidence intervals from multivariable linear regression models for relative tremor improvement (left) and stimulation-induced ataxia (right). For tremor improvement, no predictor reached statistical significance (adjusted R² = 0.07, *p* = 0.1). The ataxia model (adjusted R² = 0.29, *p* < 0.001) shows significant positive associations with stimulation amplitude and age at surgery, and significant negative associations with baseline tremor severity and stimulation frequency, while pulse width shows a non-significant positive trend. Notably, baseline ataxia was not associated with ataxia worsening

Put together, tremor improvement was not strongly driven by any standard demographic, clinical or stimulation characteristic, though some trends emerged. Conversely, stimulation-induced ataxia was related to higher stimulation amplitude and older age at surgery, and showed further negative association with baseline tremor severity and stimulation frequency and was not associated with preoperative gait ataxia scores. (Fig. 2)

### Anatomical segregation of therapeutic and adverse effects

All active contacts were centred in the subthalamic area (Fig. 4). The spatial distribution of outcome-related VTAs revealed a clear dissociation between tremor control and gait ataxia. Greater tremor reduction was associated with more anterior and superior stimulation. In contrast, more posterior stimulation was linked to less tremor improvement, yielding a tremor “sweet spot” defined as the region where stimulation was significantly associated with increased tremor improvement, located just dorsal to the medial STN (centre of gravity (COG), ACPC: −13.0, − 0.9, − 2.9; x, y, z).

**Fig. 3:**
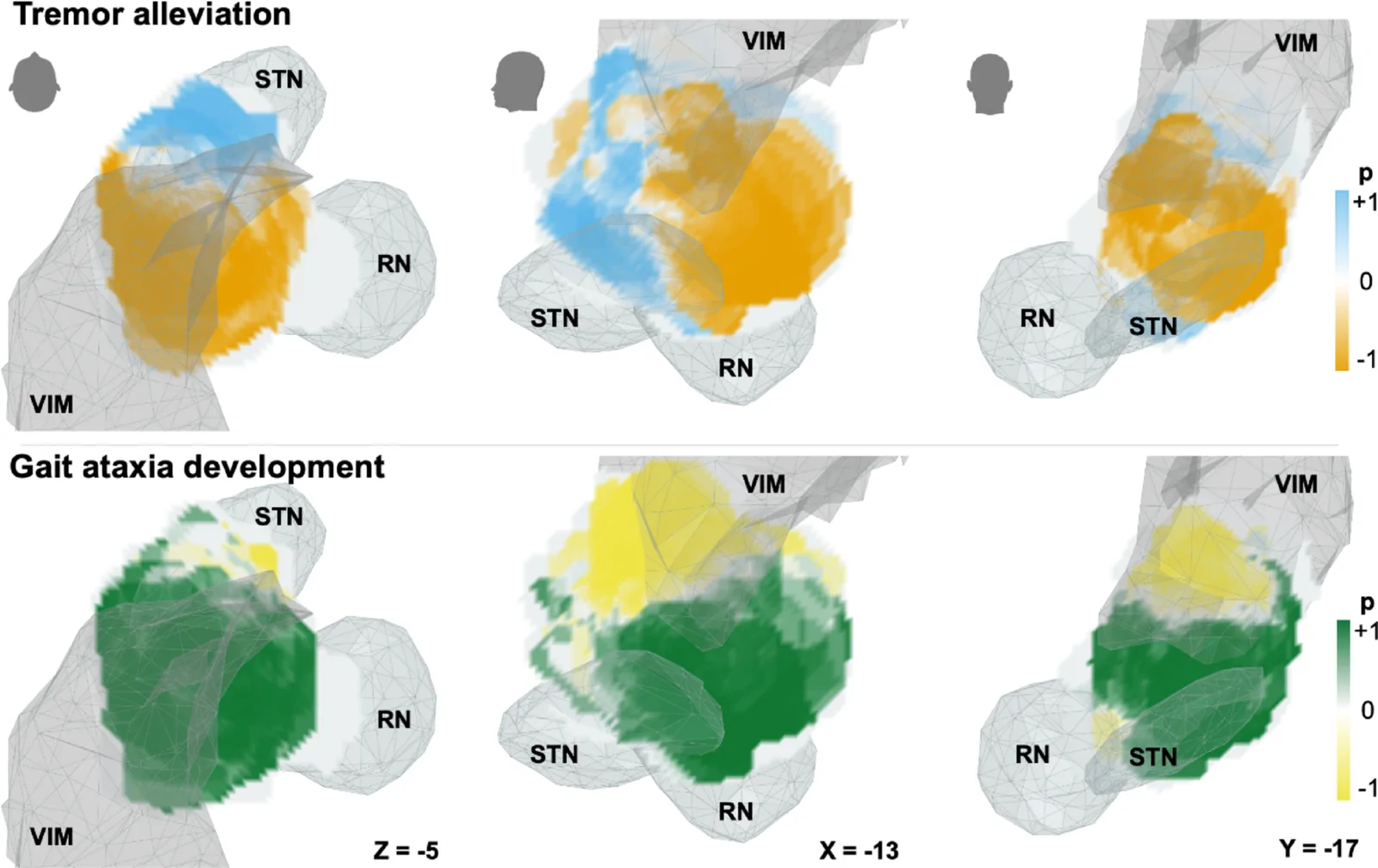
Spatial distribution of outcome-related heatmaps (signed p-maps) for tremor (top row) and gait ataxia (bottom row). Axial, sagittal, and coronal views are shown, with representative slices selected near the centre of the stimulated area. Anatomical nuclei, including the red nucleus (RN), subthalamic nucleus (STN), and ventral intermediate nucleus of the thalamus (VIM), are included for reference.For tremor (top row), colours range from blue to orange, indicating greater to lesser tremor improvement, respectively. Greater tremor reduction was observed with more anterior and superior stimulation, whereas more posterior regions are associated with less tremor benefit. For gait ataxia (bottom row), colours range from green to yellow, indicating higher to lower risk of gait ataxia, respectively. The highest probability of ataxic side effects occurs in regions posteromedial to the STN, while more superior regions within the VIM are associated with lower ataxia risk.

In contrast, the probability of gait ataxia was highest for VTAs centred posteromedial to the STN, while more superior regions within the VIM were associated with less ataxia. This pattern defined a gait-ataxia “sour spot” as the region where stimulation was most strongly associated with worsening or development of gait ataxia, posteromedial of the STN (COG, ACPC: −12.9, − 6.2, − 3.3) (Fig. 3).

In keeping with the spatial patterns observed in the single-outcome heat maps, the NPC analysis identified two favourable stimulation targets showing a significant convergence of beneficial tremor effects and low ataxia risk. One in the anterior VIM (COG, ACPC − 12.3, − 4.2, 3.1) and a second in the superior STN and its vicinity (COG, ACPC − 13.3, − 0.9, − 3.7 (Fig. 6).

**Fig. 4:**
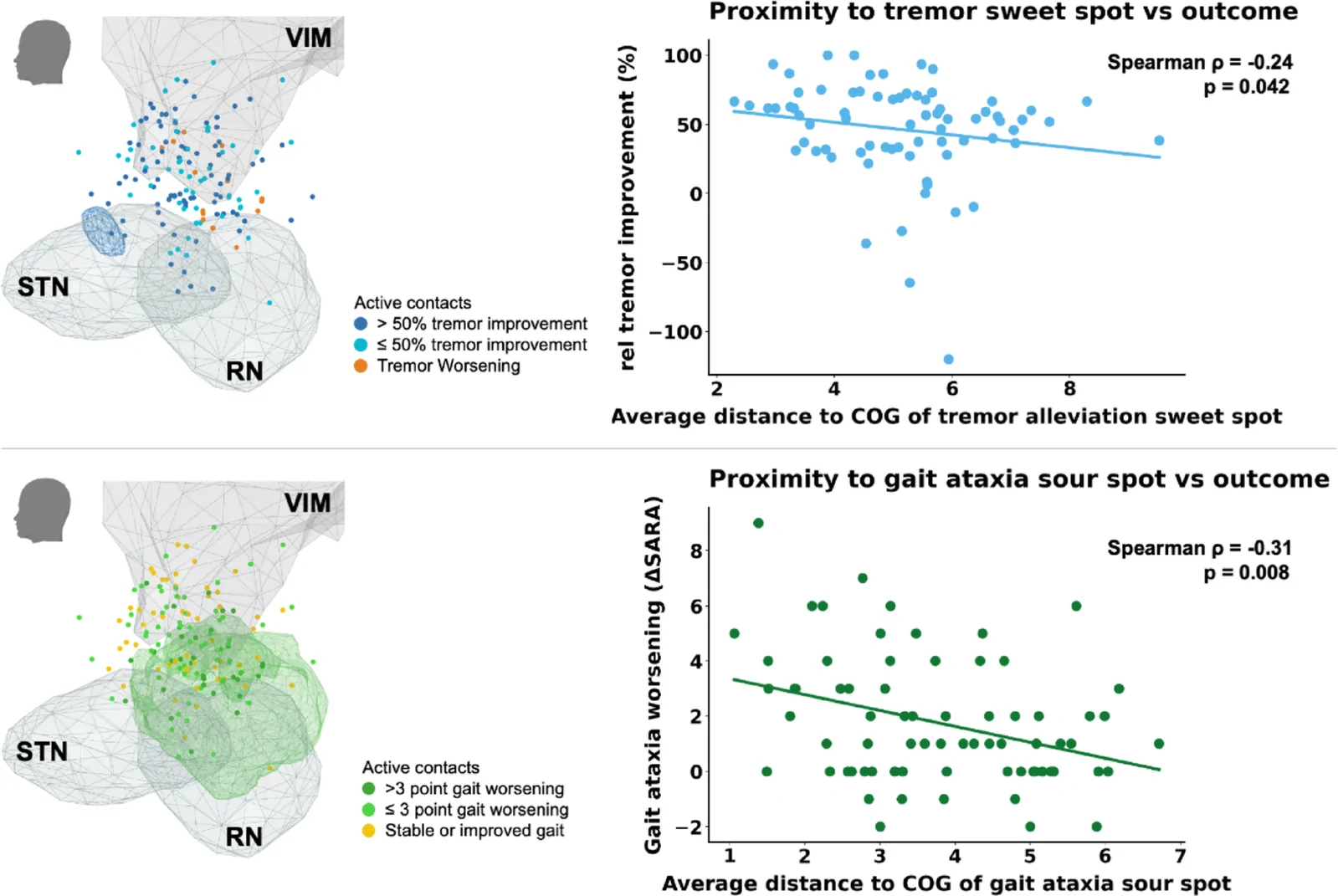
Spatial coupling between stimulation sites and tremor and gait effects. For each patient, the mean Euclidean distance between t and right active contact and the COGs of the tremor sweet spot and gait-ataxia sour spot was correlated with clinical outcome. Top row (tremor): Left, sagittal view the lefshowing the tremor sweet-spot volume (dark blue) and active contacts for both hemispheres. Points are coloured by tremor outcome: ≥50% improvement (dark blue), 0–50% improvement (light blue), and tremor worsening (orange). Right, Spearman correlation between distance to the tremor sweet-spot COG and tremor outcome (ρ = −0.24, p = 0.042), indicating that closer proximity to the tremor sweet spot was associated with greater tremor improvement.Bottom row (gait ataxia): Left, sagittal view showing the gait-ataxia sour-spot volume (dark green) and active contacts. Points are coloured by SARA outcome: >3-point worsening (dark green), ≤3-point worsening (light green), and stable or improved gait (yellow). Right, Spearman correlation between distance to the gait-ataxia sour-spot COG and gait outcome (ρ = −0.31, p = 0.008), indicating that closer proximity to the sour spot was associated with a higher likelihood of gait ataxia.

When correlating each patient’s mean (left/right-averaged) Euclidean distance between the active contact and the COG of the tremor sweet spot or gait-ataxia sour spot with the observed clinical outcome, greater distance was associated with less tremor improvement and a lower probability of developing gait ataxia. These associations were significant for both tremor (Spearman ρ = −0.24, *p* = 0.042) and gait ataxia (ρ = −0.31, *p* = 0.008), providing internal validation of the identified sweet- and sour-spot locations (Fig. 4).

In summary, tremor benefit localized to more anterior stimulation, whereas more posterior and inferior stimulation increased the risk of ataxic side effects and reduced tremor benefit. Although simple proximity to the corresponding sweet and sour spots showed the expected direction of association, the explained variance was modest, motivating the subsequent voxel-wise probabilistic modelling.

## Probabilistic mapping and building of a predictive model

**Fig. 5:**
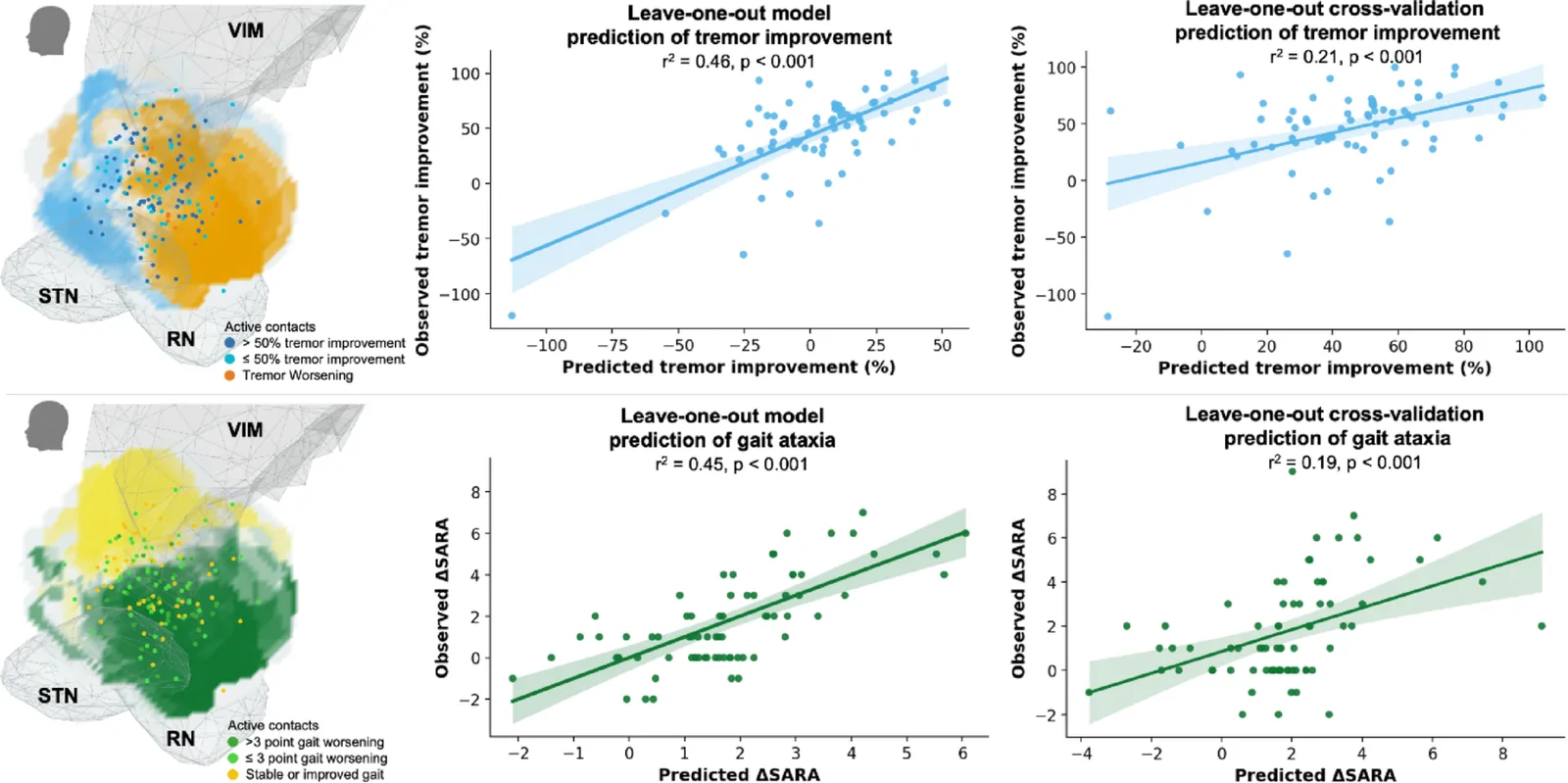
Probabilistic prediction models of tremor and gait ataxia outcomes. Linear models were trained using voxel-wise VTA interference histograms under leave-one-out (LOO) settings. Top row (tremor): Left: sagittal 3D rendering of the probabilistic tremor heat map (blue→orange, greater→lesser tremor improvement), with all active DBS contacts projected onto the heatmap slice and colored by clinical outcome (≥50% improvement, 0–50% improvement, worsening). Middle: model fit of the probabilistic tremor prediction model (r² = 0.46, p < 0.001). Right: LOO cross-validated prediction of tremor improvement, demonstrating preserved out-of-sample performance (r² = 0.21, p < 0.001). Bottom row (gait ataxia): Left: sagittal 3D rendering of the probabilistic gait-ataxia heat map (green→yellow, higher→lower ataxia probability), with all active DBS contacts projected onto the slice and colored by SARA outcome (>3-point worsening, ≤3-point worsening, stable/improved). Middle: model fit of the probabilistic gait-ataxia prediction model (r² = 0.45, p < 0.001). Right: LOO cross-validated prediction of gait outcome from VTA interference patterns, showing significant out-of-sample performance (r² = 0.19, p < 0.001). Error bars indicate 95% confidence intervals. Heat-map colour scales and spatial interpretation see Fig. 3.

Probabilistic prediction models based on individual VTA interference histograms demonstrated robust performance. For tremor, the initial leave-one-out (LOO) model showed good fit (r² = 0.46, *p* < 0.001) to the data, which remained predictive under LOO cross-validation to control for overfitting (r² = 0.21, *p* < 0.001). For gait ataxia, the corresponding model achieved a similarly strong initial fit (r² = 0.45, *p* < 0.001) and preserved predictive power in LOO cross-validation (r² = 0.19, *p* < 0.001). (Fig. 5)

As several demographic, clinical, and stimulation parameters were associated with the occurrence of gait ataxia, we augmented the linear model by including age at onset, baseline tremor severity, stimulation amplitude, and stimulation frequency as covariates. The gait-ataxia model remained highly significant and showed improved explanatory power even after multivariable adjustment in the leave-one-out cross-validation (adjusted *R*² = 0.49, *p* < 0.0001). For completeness, we applied the same covariate-adjusted framework to tremor outcome, yielding a comparable model fit (adjusted *R*² = 0.37, *p* < 0.0001).

Together, these analyses demonstrate that probabilistic mapping produced significant and reproducible predictive models for both tremor and gait ataxia, accounting for 21% and 49% of the variance in the final leave-one-out–validated models, respectively. In this framework, gait ataxia showed especially strong explanatory power. Notably, probabilistic mapping models outperformed the simpler Euclidean-distance approach to the “sweet” and “sour” spots, indicating that voxel-wise probabilistic mapping captures clinically relevant spatial information beyond proximity to a single centroid.

For both multivariate models, variance inflation factors for the spatial predictors were low (VIF = 1.89 and 1.91), indicating minimal multicollinearity of covariates.

**Fig. 6:**
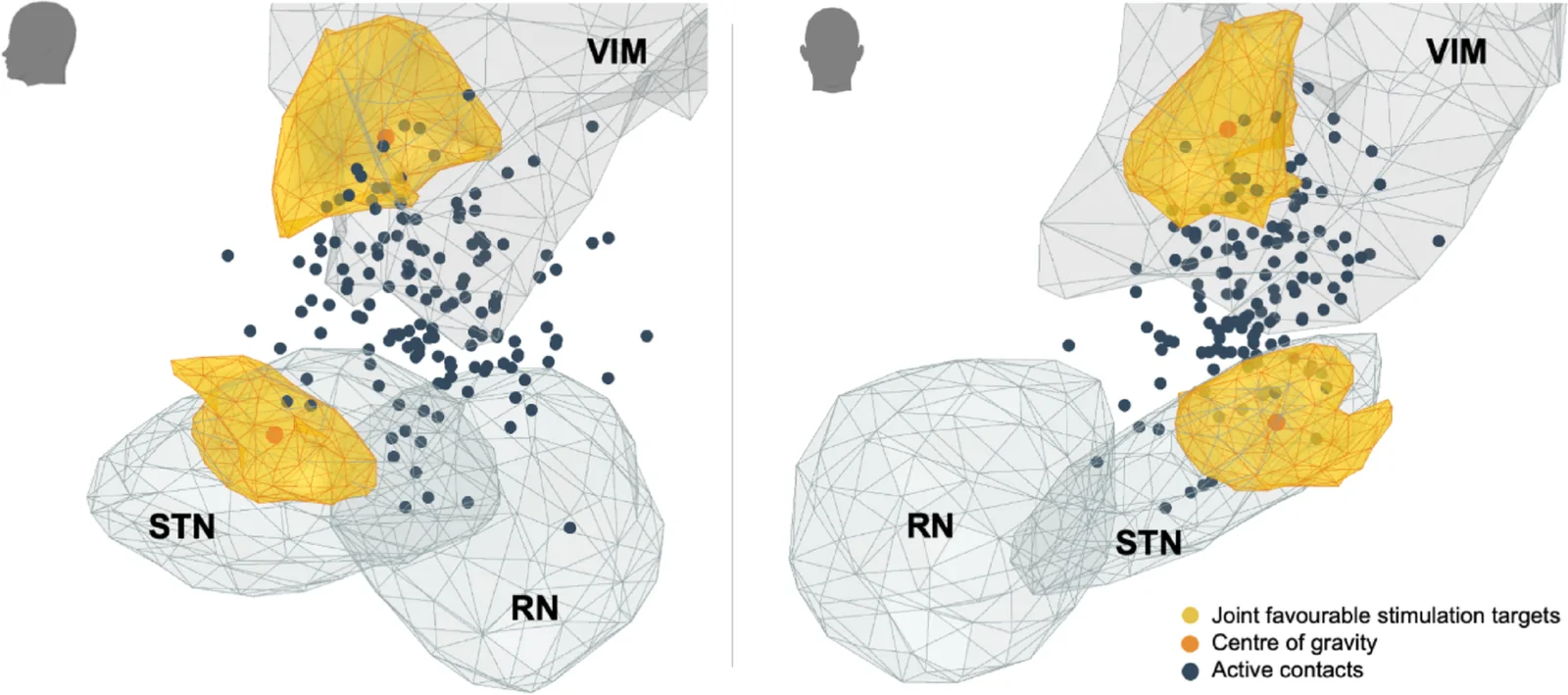
Joint favourable outcome stimulation targets in non-parametric combination analysis. Sagittal (left) and coronal (right) views including significant target clusters and centres of gravity in the anterior ventral intermediate nucleus of the thalamus (VIM: ACPC −12.3, −4.2, 3.1) and the subthalamic nucleus and its vicinity (STN: ACPC −13.3, −0.9, −3.7) as well as all active DBS contacts.

## Discussion

In this retrospective study of 73 ET patients from two DBS centres, we combined clinical and stimulation variables with probabilistic mapping of individual VTAs to build predictive models for tremor benefit and gait ataxia. We found four main results: (i) DBS reduced tremor severity on average by 46%, with 77% of patients free of functionally relevant tremor after 12 months but 44% experiencing clinically relevant gait ataxia; (ii) tremor outcome was not related to demographic or baseline clinical conditions or stimulation parameters, but showed a clear spatial pattern, with more anterior stimulation of the subthalamic area associated with greater tremor improvement, and the final model explained about 21% of the variance in tremor outcome; (iii) development of gait ataxia was related to higher stimulation amplitude, higher age at surgery, lower stimulation frequency and lower baseline tremor, and was linked to more posterior and inferior stimulation, with a final combined model explaining about 49% of the variance in gait ataxia; and (iv) together, these findings demonstrate that probabilistic mapping can be used to derive predictive models for both therapeutic benefit and stimulation-induced side effects in ET and could plausibly aid clinical programming in the future.

### Clinical landscape and phenotyping challenges

Although this study again illustrates the therapeutic efficacy of DBS in ET, stimulation-induced adverse effects remain a major limitation, with 44% of our cohort exhibiting noticeable gait disturbances, similar to previous reports (Chiu et al. 2020). Importantly, gait ataxia is only part of a broader spectrum of stimulation-evoked side effects in these patients, which additionally include dysarthria, paresthesias, muscle spasms and appendicular dysmetria (Levy et al. 2020; Kim et al. 2021). A major constraint of the existing literature is the heterogeneity in the classification of stimulation-induced side effects. The umbrella term “ataxia” frequently encompasses divergent symptoms with appendicular dysmetria, saccadic abnormalities, dysarthria and wide-based gait all subsumed under this label (Chiu et al. 2020). Even within gait-focused assessments, studies report divergent phenotypes, ranging from predominantly appendicular dysmetria to mainly axial postural and gait difficulties (Groppa et al. 2014; Reich et al. 2016). Such heterogeneity complicates comparisons across studies and is likely a factor that hindered previous attempts at anatomical and causal dissociation (Levy et al. 2020). To address this, during the specific rating of the standardized video recordings for this study, we deliberately restricted our definition to gait-dominant axial ataxia using SARA items 1–3, focusing on a clinically coherent phenotype.

### Determinants of tremor control

The magnitude of tremor suppression observed in our cohort was comparable to that reported in previous studies and, consistent with prior work, showed no substantial relationship with individual DBS parameters (Lu et al. 2020; Bogdan et al. 2020). Instead, the anatomical location of the stimulation emerged as the primary determinant of tremor benefit. Probabilistic mapping in our cohort revealed greater tremor improvement with more anterior and inferior stimulation, identifying an optimal zone just dorsal to the medial STN, consistent with prior mapping studies and a randomized controlled trial (Levy et al. 2020; Dembek et al. 2017; Kvernmo et al. 2022). Moreover the NPC analysis identified this imitate vicinity of the STN as well as the anterior VIM as an additional target region in the joint outcome analysis. These converging findings support a tract-based model in which DBS alleviates tremor by modulating the dentato–rubro–thalamic tract (DRT) rather than anatomical nucleus. (Fox and Deuschl 2022). The DRT courses through the anterior portion of the stimulated region, and several studies have shown that proximity to this tract predicts tremor outcome (Groppa et al. 2014; Dembek et al. 2020). The improved tremor benefit observed with more anterior and inferior stimulation may therefor reflect better anatomical accessibility of the DRT (Kvernmo et al. 2022). Collectively, these findings suggest that refining tract-oriented targeting and shaping stimulation fields may be the most effective strategy for improving tremor control. Interestingly, the data-driven joint-outcome NPC analysis identified both the immediate vicinity of the STN and the anterior VIM as favourable regions when considering tremor benefit and gait ataxia risk though the study design did not allow for a direct comparison of both targets. The model performance, though promising for future computer guided programming attempts, is likely still constrained by the limitations of clinical rating scales compared with more objective kinematic measures (Lange et al. 2025a; Friedrich et al. 2024). and by the fact that different tremor aetiologies or components (e.g. postural vs. intention tremor) may rely on partially distinct network mechanisms (Muthuraman et al. 2018; Dembek et al. 2017).

### Determinants of stimulation induced gait ataxia

Contrasting with tremor improvement, stimulation-induced gait ataxia showed clear associations with demographic, clinical and stimulation factors. Higher age at surgery and lower baseline tremor severity were linked to greater gait ataxia after adjustment for covariates. An association with higher age may point towards a reduced compensatory reserve in this progressive disorder. Moreover, as gait ataxia may emerge only after months or years of continuous stimulation, this pattern would be compatible with the hypothesis that axial gait ataxia is mediated by a secondary failure of compensatory mechanisms rather than an immediate stimulation effect (Reich et al. 2016; Kim et al. 2021).

These findings contrast previous work proposing shorter disease duration as a risk factor for ataxia, hypothesised to reflect more aggressive subtypes, but comparability to this study is limited as the term “ataxia” included several heterogeneous cerebellar symptoms (Chiu et al. 2020).

In terms of stimulation parameters, gait ataxia was strongly associated with higher stimulation amplitude and showed a positive, though non-significant, trend with pulse width, in line with previous reports (Reich et al. 2016; Contarino et al. 2017). These findings reveal a critical clinical programming asymmetry with tremor largely amplitude-independent, whereas gait ataxia increased with higher amplitudes, so escalating stimulation settings to improve tremor may directly increase the risk of disabling gait disturbance. Interestingly, lower stimulation frequency was also associated with increased ataxia in the multivariable model. Although this finding should be interpreted cautiously given the retrospective design and the fact that most frequencies in our cohort should still be considered high frequency (≥ 120 Hz), it contrasts with a small proof-of-concept work suggesting that lowering frequency may improve balance (Ramirez-Zamora et al. 2016). As the changes in frequency in that study also yielded a lower total electrical energy delivered, the primary driver of the observed changes in it remains unclear.

In the probabilistic mapping Gait ataxia was most likely when stimulation occurred in posteromedial and inferior regions of the subthalamic area, closely matching studies implicating the posterior subthalamic area, a more inferior stimulation of the PSA and regions adjacent to the red nucleus in gait dysfunction (Fytagoridis et al. 2013; Reich et al. 2016; Groppa et al. 2014). Conversely, stimulation fields confined to more superior thalamic regions, such as the caudal VIM, were associated with substantially lower risk (Kim et al. 2021).

Interestingly, although such reports are less common, the pattern also overlaps with PD-DBS sites linked to stimulation-related gait ataxia, suggesting a possible location-dependent effect rather than a strictly disease-specific mechanism (Farris and Giroux 2016). As such, previously proposed mechanisms involving the red nucleus or cerebello-/spinothalamic pathways like fastigio-bulbar projections remain plausible (Groppa et al. 2014; Reich et al. 2016). Our data additionally suggest that gait ataxia risk is compounded by patient-specific vulnerability and stimulation settings, consistent with a proposed chronic maladaptive recruitment of these gait-relevant pathways or failed compensation during chronic stimulation. The strong LOOCV predictability, together with low VIFs, suggests that anatomical and clinical predictors contribute largely independently. Although speculative, this framework could also explain the delayed-onset of these gait symptoms after months to years of chronic stimulation (Reich et al. 2016; Kim et al. 2021), consistent with a loss of compensation. A prospective model would be particularly relevant for such delayed-onset gait ataxia, as it could allow early identification and potentially avoidance of high-risk stimulation parameters already during the programming phase. However, further validation and disentanglement will require future prospective longitudinal and network studies.

### Local Probabilistic mapping approaches

Probabilistic mapping provides a hypothesis free, data-driven approach, leveraging stimulation and outcome data from many patients to model how the full 3D stimulation field interacts with outcome-relevant anatomy. By linking spatial dose–effect relationships to clinical benefit and side effects, it directly yields testable pathophysiological hypotheses about the circuits underlying both therapeutic and adverse effects. The probabilistic models in our study proved significant even in a leave on out cross validation underscoring the value of such analysis leveraging models of locally stimulated areas. Such models have been increasingly used to model and predict clinical outcome in PD and Dystonia (Reich et al. 2019, 2022; Nickl et al. 2025; Navratil et al. 2025; Lange et al. 2025b), with first promising results using them as basis for in silico optimized reprogramming of patients (Lange et al. 2023).

However, precisely localizing stimulation-induced effects during DBS of the (sub)thalamic region for ET remains especially challenging (Levy et al. 2020; Dembek et al. 2017). Beyond the mentioned heterogeneous symptom characterization, the dense convergence of pathways in this region leads to overlapping spatial clusters for tremor improvement and side effects, limiting anatomical dissociation (Levy et al. 2020; Dembek et al. 2017). In addition, axon-specific susceptibility to stimulation or “chronaxie”, involving properties such as diameter, orientation and distance to the electrode, can result in divergent effects from similar contact locations (Reich et al. 2015; Choe et al. 2018; Contarino et al. 2017). Although these clinical effects are widely acknowledged, current biophysical models still diverge from clinical observations, reflecting incomplete understanding of pathway-specific activation during DBS (Anderson et al. 2020). Our findings concerning the influence of frequency warrant additional consideration as it highlights the necessity to considerer frequency-dependent mechanisms in future studies especially as most VTA models do not consider this yet.

### Limitations

The retrospective design of our study limits causal inference, as the observed associations reflect averaged effects rather than responses to a controlled perturbation. However, it enabled inclusion of 73 patients, substantially larger than typical prospective DBS studies, and the variance explained by both the ataxia and tremor models, even under LOOCV, supports the robustness of the findings. Second, the retrospective setting precluded the deep prospective phenotyping that would ideally be required to anatomically dissociate stimulation-induced symptoms within the stimulated region (Levy et al. 2020). Still, restricting our analyses to gait- and stance-related items during the rating of standardized video recordings for this study increases confidence in symptom specificity. Third, as no postoperative DBS-OFF assessment was available, we cannot definitively separate stimulation-induced gait ataxia from possible progression of cerebellar symptoms in ET-plus(Fasano et al. 2010; Reich et al. 2016). However, given the relatively short 12-month follow-up, major disease progression alone is less likely to account for the observed gait worsening. Consistent with this, we did not observe an association between disease duration but conversely, gait ataxia was strongly correlated with both stimulation parameters and spatial stimulation location, supporting a predominantly stimulation-related contribution to the observed gait worsening. Fourth, the cluster-level inference threshold in the sweetspot analysis is relatively liberal (*p* = 0.05) and the resulting sweet/sour spots should therefore be interpreted cautiously though this does not affect the main findings of this study which are based on the broader spatial distribution and model of the underlying probabilistic heat maps. Finally, a possible confounder may underlie the association of higher stimulation amplitude and gait ataxia as it could reflect typical clinical attempts to compensate poor tremor performance with increased amplitude.

### Conclusion

In this large retrospective ET cohort, tremor benefit was primarily determined by a more ventral stimulation location, enabling a predictive spatial model of therapeutic response. Additionally, a distinct risk profile for stimulation-induced gait ataxia emerged, with higher stimulation amplitudes and older age as well as a more posterior–inferior stimulation. Together, these findings suggest that both benefit and side-effect risk are meaningfully predictable with probabilistic mapping approaches, providing a framework for more informed targeting and parameter selection in DBS programming. To optimize overall clinical stimulation outcome, VTA shaping toward more anterior regions of the subthalamic area, use of the lowest effective amplitude, and higher stimulation frequencies should be considered.

## Supplementary Information

Below is the link to the electronic supplementary material.

Supplementary Material 1

## Data Availability

Individual Patient Data are available upon reasonable request pending institutional approval.
