# Supplementary material for: Probabilistic mapping of tremor control and gait ataxia risk in deep brain stimulation
Source: J Neural Transm (Vienna). 2026 Mar 26;133(8):1963–75. doi: 10.1007/s00702-026-03137-9 (PMC13545145; doi:10.1007/s00702-026-03137-9)
Supplement: Supplementary file 1 [file 702_2026_3137_MOESM1_ESM.docx]

# Supplementary Material

| **Patient ID** | | **Age at surgery**  **[yrs]** | **disease duration at surgery**  **[yrs]** | **TRS Stim-PRE/ON** | **SARA Stim-PRE/ON** | **Amplitude  right//left**  **[mA]** | **Pulsewidth  right//left**  **[µs]** | **Frequency  right//left**  **[Hz]** | **Comment** |
| --- | --- | --- | --- | --- | --- | --- | --- | --- | --- |
| **WUE_01** | 81 | | 20 | 12/5 | 0/7 | 1,8//2,3 | 60//60 | 180//180 |  |
| **WUE_02** | 71 | | 9 | 17/28 | 1/10 | 3,5/3,0//3,5/2,5 | 60//60 | 120//120 | Interleaving |
| **WUE_03** | 59 | | 30 | 16/11 | 0/5 | 3,1//4,8 | 60//60 | 180//180 |  |
| **WUE_04** | 66 | | 25 | 13/5 | 4/9 | 3,4//2,8 | 60//60 | 130//130 |  |
| **WUE_05** | 72 | | 67 | 10/3 | 0/3 | 3,6//3,1 | 30//30 | 180//180 |  |
| **WUE_06** | 62 | | 22 | 15/1 | 0/0 | 3//1,7 | 60//60 | 180//180 |  |
| **WUE_07** | 63 | | 58 | 15/0 | 0/0 | 0,8//1,2 | 60//60 | 140//140 |  |
| **WUE_08** | 55 | | 40 | 10/1 | 1/0 | //2,5 | 60//60 | 130//130 |  |
| **WUE_09** | 17 | | 10 | 15/2 | 1/1 | 2,3//2 | 90//60 | 130//130 |  |
| **WUE_10** | 66 | | 35 | 19/8 | 0/0 | 3//2,5 | 60//60 | 160//160 |  |
| **WUE_11** | 75 | | 10 | 20/5 | 3/4 | 1//2 | 60//60 | 179//179 |  |
| **WUE_12** | 66 | | 61 | 24/11 | 2/6 | 1,0//3,0/2,0 | 60//60 | 125//125 | Interleaving |
| **WUE_13** | 68 | | 48 | 23/18 | 1/5 | 1,5//2,8 | 60//60 | 160//160 |  |
| **WUE_14** | 56 | | 11 | 26/10 | 2/0 | 2//2 | 60//60 | 179//179 |  |
| **WUE_15** | 62 | | 32 | 19/14 | 0/3 | 2,3//3 | 60//60 | 174//174 |  |
| **WUE_16** | 66 | | 53 | 24/7 | 1/2 | 2,5//1 | 60//60 | 180//180 |  |
| **WUE_17** | 74 | | 19 | 26/7 | 0/0 | 3,3//4 | 60//60 | 180//180 |  |
| **WUE_18** | 57 | | 43 | 16/7 | 0/0 | 1,5//1,1 | 60//60 | 180//180 |  |
| **WUE_19** | 52 | | 30 | 15/1 | 0/0 | 2,8//2,3 | 60//60 | 180//180 |  |
| **WUE_20** | 51 | | 10 | 11/0 | 0/0 | 2,3//2,9 | 60//60 | 180//180 |  |
| **WUE_21** | 68 | | 63 | 22/8 | 1/2 | 2,6//2,4 | 30//30 | 180//180 |  |
| **WUE_22** | 60 | | 12 | 19/5 | 3/1 | 2,2//2 | 30//30 | 200//200 |  |
| **KI_01** | 82 | | 30 | 26/7 | 1/2 | 2,6//2,6 | 90//60 | 130//130 |  |
| **KI_02** | 43 | | 29 | 12/8 | 0/1 | 1//2,2 | 90//90 | 130//130 |  |
| **KI_03** | 73 | | 22 | 15/33 | 0/4 | 2,3//2,1 | 60//60 | 180//180 |  |
| **KI_04** | 71 | | 54 | 22/28 | 2/4 | 4,3//3,2 | 60//60 | 180//180 |  |
| **KI_05** | 52 | | 49 | 19/12 | 0/2 | 1,8//1,7 | 90//60 | 160//160 |  |
| **KI_06** | 64 | | 10 | 15/8 | 1/4 | 1,2//2,2 | 90//90 | 160//160 |  |
| **KI_07** | 70 | | 57 | 25/18 | 4/4 | 1,6//1,8 | 90//90 | 160//160 |  |
| **KI_08** | 68 | | 30 | 24/15 | 0/6 | 4,9//5,7 | 60//60 | 180//180 |  |
| **KI_09** | 42 | | 32 | 12/4 | 0/0 | 4//2,5 | 60//60 | 210//210 |  |
| **KI_10** | 79 | | 65 | 47/15 | 4/3 | 1//1 | 60//60 | 130//130 |  |
| **KI_11** | 72 | | 57 | 35/16 | 4/9 | 2,5//3,3 | 60//60 | 130//130 |  |
| **KI_12** | 78 | | 31 | 26/10 | 2/4 | 2,5//0,8/1,0 | 90//90 | 120//120 | Interleaving |
| **KI_13** | 66 | | 8 | 20/10 | 2/4 | 2,1//2,2 | 60//60 | 130//130 |  |
| **KI_14** | 68 | | 20 | 25/15 | 3/4 | 1,6//3,3 | 60//60 | 130//130 |  |
| **KI_15** | 68 | | 25 | 10/5 | 0/3 | 2//2 | 60//60 | 130//130 |  |
| **KI_16** | 66 | | 23 | 26/17 | 0/1 | 2//1,7 | 60//60 | 130//130 |  |
| **KI_17** | 69 | | 34 | 27/19 | 0/0 | 2,3//3 | 60//60 | 130//130 |  |
| **KI_18** | 60 | | 11 | 52/24 | 3/4 | 2,8//2 | 120//60 | 130//130 |  |
| **KI_19** | 71 | | 11 | 15/15 | 0/4 | 3,7//3,5 | 60//60 | 130//130 |  |
| **KI_20** | 46 | | 46 | 44/21 | 1/2 | 0,9//0,5 | 60//60 | 130//130 |  |
| **KI_21** | 72 | | 27 | 25/10 | 3/4 | 2,9/1,4//2,0/1,1 | 60//60 | 125//125 | Interleaving |
| **KI_22** | 68 | | 35 | 26/18 | 0/2 | 2,4//2,5 | 60//60 | 130//130 |  |
| **KI_23** | 68 | | 9 | 32/30 | 1/4 | 2,9//2,7 | 90//60 | 160//160 |  |
| **KI_24** | 69 | | 28 | 28/4 | 0/1 | 1,3//1,4 | 90//90 | 130//130 |  |
| **KI_25** | 57 | | 36 | 23/10 | 1/1 | 1,6//3 | 60//60 | 185//185 |  |
| **KI_26** | 78 | | 50 | 32/12 | 3/4 | 1,7//1,5 | 60//60 | 130//130 |  |
| **KI_27** | 83 | | 78 | 26/7 | 3/9 | 2,8//2,6 | 90//90 | 130//130 |  |
| **KI_28** | 69 | | 26 | 26/8 | 0/0 | 1,1//1,6 | 60//60 | 130//130 |  |
| **KI_29** | 71 | | 19 | 22/9 | 1/0 | 2,1//1,5 | 60//60 | 160//160 |  |
| **KI_30** | 62 | | 48 | 26/16 | 3/5 | 1,2/1,6//0,6/0,9 | 60//60 | 125//125 | Interleaving |
| **KI_31** | 68 | | 33 | 39/26 | 1/1 | 2,1//3,1 | 120//90 | 180//180 |  |
| **KI_32** | 56 | | 41 | 15/5 | 0/0 | 1,7//1,5 | 60//60 | 160//160 |  |
| **KI_33** | 69 | | 12 | 22/30 | 0/6 | 3,1//3,2 | 60//90 | 180//180 |  |
| **KI_34** | 68 | | 9 | 27/12 | 0/0 | 2,7//2,5 | 60//60 | 130//130 |  |
| **KI_35** | 69 | | 12 | 28/19 | 5/7 | 2,7//3,6 | 60//60 | 130//130 |  |
| **KI_36** | 67 | | 55 | 22/3 | 0/2 | 3,8//3,8 | 60//60 | 180//180 |  |
| **KI_37** | 67 | | 31 | 25/17 | 2/4 | 7,1//1,3 | 90//60 | 180//180 |  |
| **KI_38** | 59 | | 5 | 22/25 | 0/4 | 3,3//1,9 | 90//90 | 210//210 |  |
| **KI_39** | 69 | | 69 | 24/15 | 2/0 | 2,5//2 | 60//60 | 130//130 |  |
| **KI_40** | 65 | | 11 | 31/34 | 1/7 | 5,6/5,2//5,5/5,1 | 60//60 | 125//125 | Interleaving |
| **KI_41** | 65 | | 40 | 22/16 | 3/5 | 1,7//1,9 | 60//60 | 130//130 |  |
| **KI_42** | 68 | | 34 | 21/13 | 2/2 | 1,5//0,5 | 60//60 | 130//130 |  |
| **KI_43** | 78 | | 41 | 35/32 | 4/5 | 3,1//2,1 | 120//90 | 230//230 |  |
| **KI_44** | 77 | | 15 | 18/7 | 3/8 | 3,5//3 | 90//60 | 180//180 |  |
| **KI_45** | 70 | | 54 | 31/10 | 0/3 | 1,1//1,9 | 60//60 | 130//130 |  |
| **KI_46** | 66 | | 58 | 27/13 | 0/0 | 3,8//3,8 | 60//60 | 130//130 |  |
| **KI_47** | 73 | | 64 | 24/13 | 1/1 | 4,9//3,9 | 60//60 | 210//210 |  |
| **KI_48** | 64 | | 50 | 11/7 | 0/4 | 3,6//2,4 | 60//90 | 180//180 |  |
| **KI_49** | 74 | | 10 | 30/14 | 0/3 | 1,8//2,3 | 60//60 | 130//130 |  |
| **KI_50** | 64 | | 35 | 21/7 | 0/0 | 3,8//1,6 | 60//60 | 150//150 |  |
| **KI_51** | 58 | | 17 | 54/15 | 3/2 | 2//1,9 | 60//60 | 130//130 |  |

**Supplementary Table 1.** **Demographic, clinical, and stimulation characteristics of the essential tremor cohort from both DBS centres Würzburg (WUE) and Kiel (KI).** Columns include patient identifiers, age at surgery, disease duration at the time of DBS implantation, clinical scores on the Fahn–Tolosa–Marin Tremor Rating Scale (FTM-TRS; items 1–9) and the Scale for the Assessment and Rating of Ataxia (SARA; items 1–3), each reported before (PRE) and after surgery (ON), as well as electrode type and stimulation parameters (amplitude, pulse width, and frequency). Interleaving stimulation was applied in patients WUE_02, WUE_12, KI_12, KI_21, KI_30, and KI_40.
